# The Statistics of Bulk Segregant Analysis Using Next Generation Sequencing

**DOI:** 10.1371/journal.pcbi.1002255

**Published:** 2011-11-03

**Authors:** Paul M. Magwene, John H. Willis, John K. Kelly

**Affiliations:** 1 Department of Biology and IGSP Center for Systems Biology, Duke University, Durham, North Carolina, United States of America; 2 Department of Biology, Duke University, Durham, North Carolina, United States of America; 3 Department of Ecology and Evolutionary Biology, University of Kansas, Lawrence, Kansas, United States of America; Cornell University, United States of America

## Abstract

We describe a statistical framework for QTL mapping using bulk segregant analysis (BSA) based on high throughput, short-read sequencing. Our proposed approach is based on a smoothed version of the standard 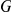 statistic, and takes into account variation in allele frequency estimates due to sampling of segregants to form bulks as well as variation introduced during the sequencing of bulks. Using simulation, we explore the impact of key experimental variables such as bulk size and sequencing coverage on the ability to detect QTLs. Counterintuitively, we find that relatively large bulks maximize the power to detect QTLs even though this implies weaker selection and less extreme allele frequency differences. Our simulation studies suggest that with large bulks and sufficient sequencing depth, the methods we propose can be used to detect even weak effect QTLs and we demonstrate the utility of this framework by application to a BSA experiment in the budding yeast *Saccharomyces cerevisiae*.

## Author Summary

Quantitative or complex phenotypes are traits that are under the control of multiple genes and environmental factors. Identifying the parts of the genome that contribute to variation in complex traits (Quantitative Trait Loci or QTLs), and ultimately the genes and alleles that are mechanistically responsible for trait variation, is a primary challenge in animal and plant breeding, population studies of human health and disease, and evolutionary genetics. In this study we describe an analytical framework that allows investigators to marry a QTL mapping approach called “bulk segregant analysis” (BSA) with high-throughput genome sequencing methodologies in order to map traits quickly, efficiently, and in a relatively inexpensive manner. This framework provides a statistical basis for analyzing BSA experiments that use next-generation sequencing and will help to accelerate the identification of QTLs in both model and non-model organisms.

## Introduction

Bulk segregant analysis (BSA; [1]) is a QTL mapping technique for identifying genomic regions containing genetic loci affecting a trait of interest. Starting with a segregating population from a genetic cross, individuals are assayed for the focal trait and two pools (bulks) of segregants are created by selecting individuals from the tails of the phenotypic distribution (other sampling designs can also be used as discussed below). Genotype frequencies are estimated for the two bulks, either via genotyping of individuals or via the creation of pooled DNA samples from which allele frequencies are estimated. Allele frequencies should be approximately equal between the two bulks in genomic regions without loci affecting the trait. Regions of the genome containing causal loci should exhibit allele frequency differences between bulks. BSA is most effective with high marker density and accurate allele frequency estimation within bulks [2]. The former was effectively addressed with the application of microarray based genotyping to BSA [3]–[8]. More recently, investigators have begun to use massively parallel sequencing methods to estimate allele frequencies for BSA studies [9]–[11], which has a number of advantages. For organisms with moderately sized genomes, next generation sequencing can provide essentially single base-pair resolution. In such cases rather than simply observing markers in linkage with causal loci the BSA-sequencing approach should allow one to observe allelic biases at the causal loci themselves. For larger genomes where high coverage of the entire genome is less practical, BSA-sequencing still has many potential advantages. For example, it does not require the design of new genotyping arrays for new crosses and may provide greater resolution than array based genotyping. Furthermore, sequencing data yields counts of alleles at polymorphic loci and thus provides a simple and intuitive way of estimating allele frequencies.

In bulk segregant studies based on high-throughput sequencing there are two sources of variation that affect allele frequency estimates. The first is variation due to the sampling of segregants that constitute the bulks themselves. This source of variation can be minimized by increasing both the size of the segregant population and the size of the bulk samples. The second source of variation is a consequence of the measurement technique used to estimate allele frequencies in the bulks. In the case of sequencing of pooled DNA samples, the sources of variation of this second type include, but are not limited to, library preparation, sequencing chemistry, sequencing coverage, post-sequencing alignment of reads, and base/allele calling algorithms. Here again, some of these sources of variation can be minimized by standardization of experimental protocols and analysis pipelines. However some of these sources of variation, particularly stochasticity in sequencing coverage, are an inherent property of short-read sequencing methods.

In this paper, we develop explicit statistical models to describe the sources of variation that should be considered in the analysis of BSA-sequencing data. We first develop test statistics based on the classic 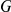 -statistic accounting for the two phase sampling inherent to BSA. We then propose an analysis pipeline for whole-genome studies and present a proof-of-concept example with data from yeast. A combination of simulation and empirical application demonstrate the utility of this analytical framework.

## Results

### Theory and Analytical Framework

#### Expected distribution of 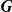 for BSA-sequencing data

Consider the experimental design with an F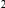 population consisting of 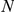 individuals, each of which is measured for a phenotype of interest. A set of 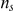 individuals from each of the tails of the distribution (low and high) are collected. DNA bulks are prepared by combining equal amounts of tissue/cells from individuals within each bulk followed by DNA extraction, or by extracting DNA from each individual and combining equal amounts. Following preparation of DNA bulks, genomic libraries are prepared and sequenced at average coverage 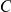 per SNP. Thus for each SNP the data is four allele counts that can be summarized in a 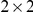 table, where 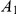 is the allele from the high parent (Table 1). The 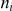-values in the table are counts of alleles not individuals. The observed allele frequency of 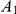 in the low bulk is 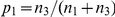; that in the high bulk is 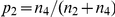. If the SNP is close to a QTL with effects in the expected direction (i.e. the ‘high allele’ increases trait values), then we expect 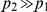.

**Table 1:** The summary of data from a single variable site.

	Low bulk	High bulk	Total
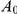	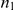	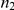	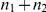
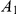	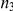	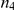	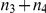
Total	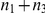	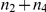	

The 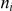 represent counts of alleles 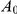 and 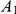 generated from sequencing of the segregant bulks.

The counts in Table 1 are determined by two levels of hierarchical of sampling. The first sample is the 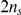 chromosomes that constitute each bulk (assuming diploid inheritance). Second, there is random variation in the number of reads per allele within each bulk due to the stochastic nature of next-generation sequencing. Let 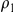 and 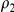 be the expected (‘true’) frequency of the high allele in each bulk. The realized frequencies (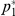, 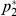) differ from 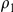 and 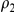 in each bulk due to binomial sampling:
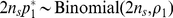
(1)

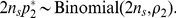
(2)If we assume that sequencing coverage is approximately Poisson, then the conditional distributions of the observed allele counts are:
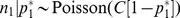
(3)

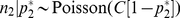
(4)

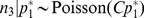
(5)

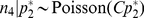
(6)


A natural statistic to characterize the data at each SNP is the standard 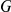 -statistic:
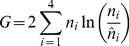
(7)where 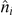 is the ‘expected value’ for count 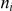. The null hypothesis is that there is no QTL close to the focal SNP. This implies the standard expected counts for a 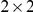 contingency table, e.g. 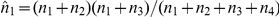. If the null hypothesis is correct, 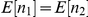 and 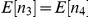. If we further assume no segregation distortion and equal (average) sequencing coverage of each bulk, then 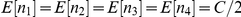. See the supplementary materials (Text S1) for a generalization that includes segregation distortion.

However, due to the hierarchical sampling scheme, the usual expectation that 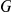 follows a 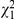 distribution (chi-square with 1 d.f.; [12]) does not hold in the present situation. The mean and variance of 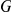 are inflated relative the 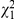 even when the null hypothesis is true (i.e. there is no QTL). Based on the arguments in Text S1 we approximate the mean and variance of 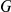 as:
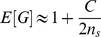
(8)

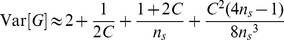
(9)These equations predict convergence on 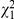 under certain parameter sets. In particular, if 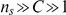, then 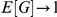 and 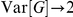, as expected from 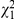.

A simulation model was used to test the accuracy of approximate equations (8) and (9). We simulated genetic data for a chromosomal region of 10 cM in recombinational length. Informative markers were uniformly distributed along this chromosome with 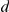 SNPs per cM. The causal locus (QTL) was located at the center of the chromosome and was thus flanked by 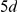 SNPs on each side. Alternative homozygotes at the QTL differ by 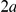 phenotypic units on average (additive gene action) and simulations of the null hypothesis (no QTL) were done with 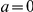. In each simulation run, we first established the genotypes and phenotypes of the 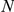 distinct F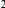 segregants. Each individual was assigned a QTL genotype according to Mendelian probabilities (0.25, 0.5, 0.25) and the phenotype was assigned as the genotypic value plus a normal deviate. Individuals were then ranked by phenotype and 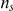 were selected from each tail. The full haplotype of these individuals was then established by working out from each allele at the QTL and allowing recombination to occur probabilistically according to the linkage map. Given the haplotypes in each bulk, we simulated an independent Poisson number for each count of Table 1 for each SNP. These data were used to calculate 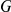 at each SNP, and also 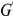 as described below, within windows around each SNP. For the latter we needed to specify a window size in centimorgans. For each parameter set, this entire procedure was repeated 10,000 times. Table 1 in Text S1 reports simulation results for the null hypothesis (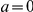) for a range of reasonable combinations of 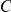 and 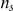. There is a close correspondence of observed means and variances of 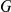 with the values predicted by equations (8) and (9). As expected, in these simulations the distribution of 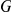 is right skewed with a mean and variance exceeding the 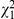 expectations.

**Figure 1:**
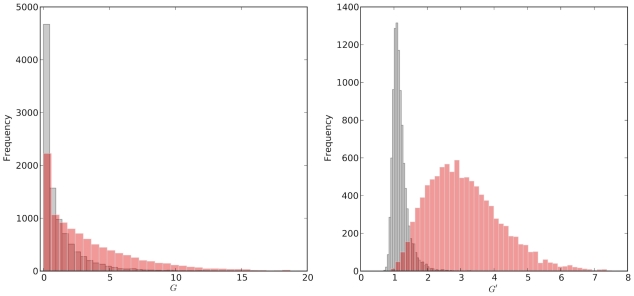
The distribution of 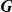 (A) and 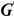 values (B) from 10,000 simulations. The gray histograms depict the observed distributions of 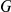 and 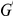 for the null case (no QTL), while the red distributions depict the distributions in the case of a weak QTL that explains 2% of the phenotypic variance.

The full distribution of 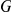 values is depicted for one parameter set (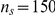, 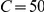) in Figure 1a. The gray histogram shows the distribution of 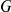 under the null hypothesis (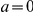) while the overlapping red histogram shows the corresponding distribution in the case of a weak QTL (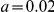). Focusing first on the null distribution, because the distribution is right skewed (mean = 1.19, variance = 2.93), if we compare this distribution to critical values of 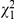 the observed false positive rate is somewhat elevated (6.98% at 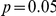; 1.98% at 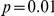). However when 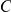 approaches 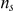 the mean and variance of 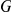 far exceed the 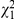 expectation and type I error rates increase dramatically. Perhaps even more problematic is the inability of 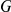 to detect a QTL based on the naïve 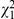 expectation. For the weak QTL case, where the QTL explains 2% of the phenotypic variance, the causal SNP is significant at a 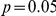 in only 34.9% of the simulations, and in only 16.8% of simulations at 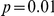. The application of the naïve 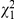 thus suffers from a lack of power.

#### 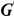, A Smoothed Version of 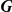


A substantial source of variation in 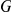 is the random margin in Table 1, 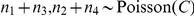. To deal with this variation we propose the use of a weighted average of 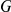 across neighboring SNPs. Averaging 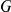 values across SNPs is sensible because the real signal of divergence in allele frequency between bulks is conserved between closely linked sites but random noise due to variable sequencing read coverage is not. We suggest the following average test statistic for each SNP:
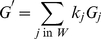
(10)where the sum includes all SNPs within the window 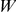 bracketing the SNP. This type of weighted moving average, where the weights are given by a kernel function, 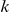, is also known as Nadaraya-Watson kernel regression [13], [14]. Nadaraya-Watson kernel regression acts as a smoothing function, with the amount of smoothing increasing with larger window size 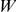 [15]. The simplest scheme for 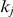 would be to give equal weight to all SNPs within 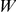 (a rectangular kernel). We opt instead to apply the tri-cube kernel fuction:
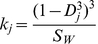
(11)where 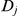 is standardized distance, with value 0 at the focal position and value 1 at the edge of the window. 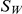 is the sum of 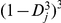 for all SNPs in 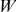. The tri-cube kernel is commonly used in local polynomial regression methods like LOESS [16] and gives greater weight to observations that are close to the focal SNP. Any other weighting kernel that decreases smoothly to 0 as 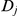 goes to 1 could be used as well. We discuss the choice of the kernel window size, 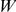, below.

A methodological issue arises when kernel smoothing is used – at the beginning or end of a data series it can produce a biased estimate because the data included in the kernel bandwidth is asymmetric. The simplest way to deal with this is to append a reflected version of the values that fall within the right half-bandwith (at the beginning of the series) and left half-bandwidth (at the end of the series), run the kernel smoother as normal, and then trim the appended values from the output.

#### Expected distribution of 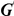 for BSA-sequencing data

The null expectation of 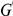 is given by equation (8). The variance of 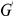 depends on the variance of individual 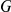 values (equation 9) and the covariance between SNPs within a window. In Text S1 we show that 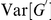 can be approximated as:
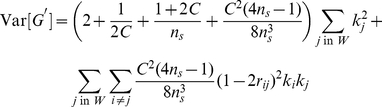
(12)where 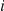 indexes all SNPs other than 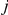 contained within the window.

Figure 1b illustrates the distribution of 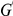 for the same parameters as Figure 1a (plus window size 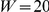 cM and SNP density 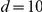 per cM). The difference between the null distributions in Figure 1a and 1b is due to the normalizing effect of averaging. The predicted mean and variance of 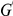 (1.17 and 0.066) are reasonably close to the observed moments (1.18 and 0.056). The distribution of 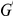 is still right skewed but the right tail can reasonably predicted from log-normal densities with parameters derived from 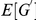 and 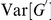 (Figure S1 and Text S2). The observed false-positive rates (using a log-normal density estimation) are: 5.14% at 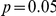 and 1.86% at 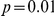). Unlike the use of the naive 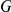 -test based on 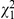, the type I error does not increase dramatically as 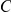 approaches 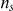. Furthermore, 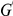 has good power to detect QTLs. For the example illustrated in Figure 1b the causal SNP is significant in 94.3% of the simulations at 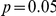, and in 88.0% and 77.2% of simulations at 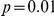 and 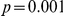 respectively.

#### Non-parametric estimation of the null distribution of 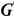


In addition to the theoretical expectations discussed above, an empirical estimate of the null distribution of 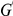 can be derived from the observed data itself. We assume that the observed data, 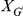, is a mixture of the null distribution (non-QTL regions) and several contaminating distributions (QTLs). As discussed above, the null distribution of 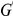 (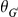) is right-skewed with a tail density reasonably predicted from a log-normal distribution, 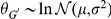. We also assume the contaminating distributions have higher means than the null distribution. Our goal is to estimate 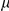 and 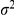 in a manner that is not unduly influenced by the contaminating distributions.

Recall that for a log-normal distribution: 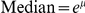 and 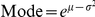 [17]. Thus if we can estimate the median and mode of 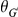 can use those to estimate 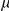 and 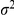. To do so we propose the folowing steps:

1. Let 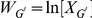

2. Let 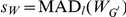, the left median absolute deviation (MAD) of 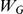 where 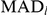 is defined as
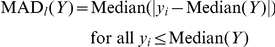

3. Use Hampel's rule [18] to identify outliers, 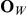, as all 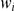 in 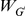 that satisfy:
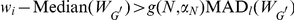
where 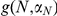 defines the limits of the outlier regions [18] and is usually taken to be 5.2 for normally distributed data.
4. Construct a trimmed data set 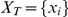 for all 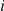 such that 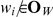

5. Let 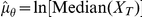 and 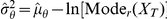 where 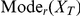 is a robust estimator of the mode for continuous variables (see [19] for several such estimators)

The logic of this procedure is as follows. The median and MAD are robust estimators of location and spread respectively [20]. In the absence of contaminating distributions 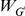 should be approximately normally distributed, and hence the median and MAD of 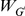 can be used as robust estimates of the mean and spread of 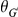 (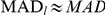 for a symmetric distribution). Hampel's rule is a commonly used procedure to identify likely outliers in a set of data based on the median and MAD; if the underlying distribution is normally distributed and 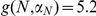 this is approximately equivalent to identifying outliers as those observations with 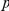-values 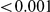 (we use a one-sided test in the procedure above). When contaminating distributions (QTLs) are present, 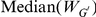 lies to the right of the true mean of the null distribution. Thus, 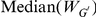 and 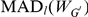 are conservative estimators of 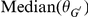 and 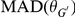. We then use Hampel's procedure to identify observations likely to be drawn from the contaminating distributions and create a trimmed data set, 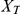, with those outlying observations removed. From the trimmed data set we estimate 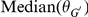 and 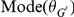.

For the null simulations in Figure 1b the observed false-positive rate estimated using this non-parametric approach are 3.18% at 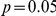 and 0.76% at 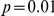. In general, the non-parameteric procedure tends to be slightly more conservative than our proposed parametric estimators but not greatly so. Because this non-parametric approach makes few distributional assumptions (other than approximate log-normality of the null distribution) it might be preferred in cases where one suspects the sampling (either of segregants or alleles) grossly violates the hierarchical model described above.

#### Choosing 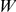


A weighted moving average is a type of low-pass filter; the larger the window size the lower the frequncy of signals that are rejected by the filter. The choice of smoothing width, 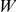, is therefore a tradeoff between filtering out high-frequency deviations in 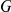 due to variable sequence coverage and SNP density and attenuating the signal of real QTLs. We want to pick a 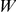 that minimizes noise while maximizing the underlying signal. The matched filter theorem [21] suggests that the filter that maximizes the signal-to-noise ratio of a symmetric signal is one which matches the shape of the signal. A simple measure of the shape of a symmetric signal is the full-width at half maximum (FWHM). The ratio of the width of the kernel to the peak FHWM (‘smoothing ratio’) is a useful metric for quantifying the effects of smoothing [22]. As a rule of thumb, using a smoothing kernel with a smoothing ratio of approximately two provides a good signal-to-noise ratio [22]. However, the matched filter may fail to distinguish multiple peaks when there are two or more signals in the input [23] as we would expect in cases of multiple QTLs with overlapping regions of elevated 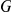. Specifically, peaks separated by less than twice the FWHM of the filter will be merged [24]. Therefore, to distinguish overlapping signals requires filters with smoothing ratios significantly smaller, perhaps as small as 0.7.

In Text S2 we derive the expected shape of 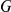 around a single causal SNP. For the case in which the causal allele is fixed in one bulk and has a frequency of 0.5 in the other bulk, the half-bandwidth (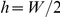) at half-maximum corresponds to 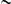
12.42 cM (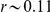). More extreme allelic biases between the bulks favor slightly smaller bandwidths, while less extreme differences favor larger bandwidths. SNP density also affects the optimal kernel bandwidth, with higher SNP density favoring narrower bandwidths. In simulations and applied to real data we have found that kernels with smoothing ratios in the range 1–1.5 produce smoothed estimators with good signal-to-noise ratios and which are neither strongly over- or undersmoothed. In terms of mapping distances this corresponds to kernels with 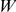 in the range 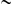
24.8–37.25 cM.

Since recombination rates vary across genomes, a given genetic distance will correspond to a range of physical distances. In terms of the choice of smoothing width, higher recombination rates favor smaller window sizes (in physical distance). If regional recombination rates are known this can be incorporated into the analysis; however the use of average chromosomal or genomic recombination rates to choose a single physical size for the smoothing window should not be problematic unless recombination rates vary widely. In such cases, one can calculate 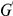 using a range of smoothing widths to explore whether peak estimates are strongly affected by over- or undersmoothing.

### Proposed Analytical Pipeline

Based on the arguments developed above, we propose the following analytical pipeline for the analysis of BSA-sequencing data sets. We assume that sequencing reads have been aligned to a reference genome where physical distances between polymorphic sites and (approximate) rates of recombination are known. We assume that all sites are biallelic. Following alignment of reads to a reference genome, per site counts of each allele are generated from the reads. Our recommended analysis pipeline for estimating QTLs is as follows:

1. For each variable site, calculate 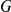 based on the observed number of reads for each allele in each of the two pools
2. At each site calculate 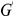 using a smoothing kernel with bandwidth 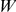 bases where 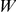 is chosen based on known or estimated rates of recombination. Bandwidths should typically correspond to genetic map distances in the range 25–40 cM.
3. Estimate parameters of the log-normal null-distribution (i.e. no QTL) of 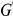, 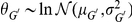, based on either theoretical expectations (equations (8) and (12 and Text S2) or using the robust empirical estimator of the null distribution inferred from the observed 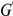.
4. Using 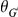 estimate 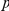-values directly using the log-normal CDF. Alternately log-transform 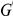 and calculate 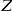 scores 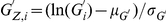 and corresponding 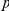-values at each site.
5. Use a false discovery rate approach (FDR; [25], [26]) to account for multiple comparisons and estimate an appropriate p-value threshold (or the corresponding 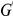 threshold) to determine sites that deviate significantly from the background null distribution
6. Define candidate QTL regions as continuous runs of significant sites

### Power Analysis

**Figure 2:**
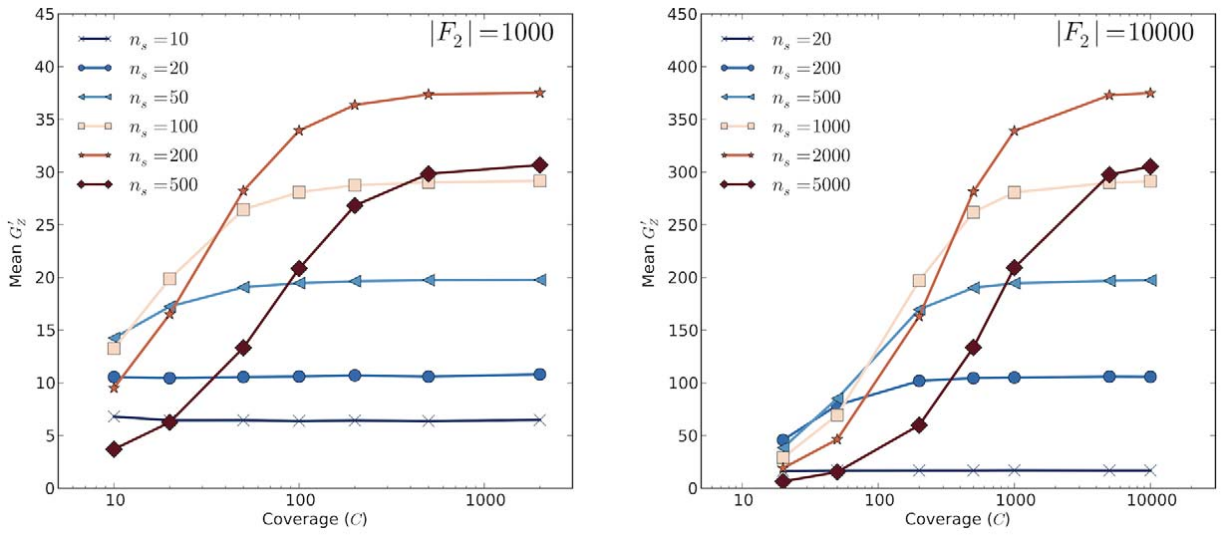
Power analysis. Average 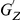 at a causal site as a function of sequencing coverage, 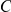, and bulk size, 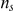, for two different F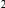 population sizes (left, 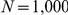; right, 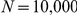). Note the difference in scales between the two figures.

We used simulations to conduct a simple power analysis of our proposed methodology. In this analysis we used the mean 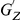 at a causal site as measure of power for given values of 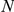, 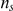, 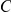, window size (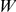), SNP density, and for different magnitudes of QTL effect on phenotype. Figure 2 summarizes results for two different values of 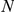, corresponding to large (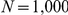) and very large (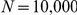) F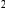 populations. We find that increasing coverage, 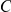, is advantageous until 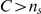, but has minimal effect beyond that. A somewhat counterintuitive result is that larger bulk size, 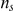, is generally beneficial as long as sequencing coverage is modest to high. This is despite the fact that larger bulks imply weaker selection for a given 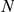 (and hence a smaller allele frequency divergence among bulks). Based on these findings we recommend bulks consisting of at least 10% and as perhaps as high as 20% of the F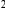 segregant population in order to maximize power to detect QTLs.

### An Application to Yeast

To demonstrate the correspondence between theory and data we here draw on a BSA-sequencing data set generated to identify loci that contribute to variation in colony morphology in the budding yeast *Saccharomyces cerevisiae* [27]. A full description and analysis of these data will appear elsewhere (Granek et al., in prep). Here, these data serve to illustrate the utility of both our theoretical framework and the associated robust estimators for data analysis.

The yeast data consist of a low and high bulk, each composed of 288 homozygous diploid segregants drawn from an F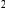 population of size 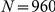 generated by sporulating a naturally heterozygous diploid strain [28]. The low bulk consists of segregants with simple colony morphology, while the high bulk consists of segregants with complex colony morphology (see [27] for a description of morphology scoring). Creation of DNA pools, sequencing, and mapping of reads is described in the Methods section. Because each segregant is homozygous, the effective number of alleles sampled for each bulk is 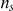 instead of 2 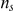. In total 44,066 polymorphic sites were analyzed with a mean interval between sites of approximately 280 bp. Below we refer to the two sequencing runs for the low bulks as 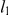 and 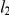, and those for the high bulks as 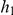 and 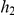. The coverage per SNP (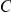) for each sequencing run was as follows: 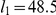, 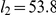, 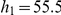, and 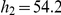. For each of the analyses below, we used a smoothing window width of 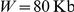 (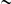
30 cM), and took the average coverage of each bulk being compared as the estimate of coverage, 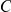.

Because there are two sequencing runs per DNA pool, variation in allele frequency estimates between sequencing runs from the same segregant bulk should be exclusively due to stochastic aspects of the sequencing reaction and primary bioinformatics analyses (base calling, read alignment). The structure of this data set is thus useful for dissecting the impact of sequencing variation on estimates of 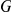 and 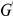, and the subsequent impact of this variability on the inference of QTL regions and peaks. We use these data to explore both the null model (no QTL; by analyzing the low-vs-low and high-vs-high comparisons) as well as the case where QTLs are expected (comparing low-vs-high bulks). In the null case, the differences in allele frequencies are subject to only one source of variation because the bulks are fixed but sequencing is variable. The non-null analyses are individually affected by both sources of variation (bulking and sequencing), but when comparing the results from comparable analyses (e.g. comparing QTL peak locations between the 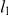-vs-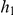 and 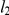-vs-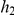 analyses), the differences are again simply a function of sequencing variation.

#### Null comparisons: Variation in 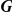 and 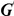 due to sequencing

The two low samples (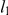 and 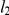) and the two high samples (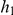 and 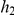) represent independent sequencing runs of the same low and high segregant bulks respectively. Using 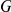 and 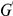 from a comparison of 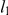 vs. 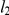 and 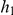 vs. 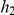 we can estimate the impact of sequencing on the variation of these statistics. When the two bulks differ only due to read number variation, there is only one source of variation, and the statistics of 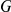 should should be approximately 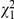 with 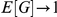 and 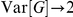. By invoking a weighted version of the central limit theorem [29], we find the distribution of 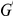 should be approximately normal with 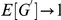 and 
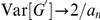
 where 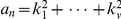, the sum of the 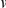 squared kernel weights in the smoothing window (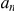 converges to 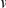 in the case of a square kernel). As illustrated in Table 2 the observed data for the null-comparisons conform well to the asymptotic expectations.

**Table 2:** Null comparisons for the yeast data set.

Comparison	Theoretical 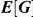 , 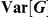	Observed 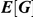 , 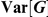	Theoretical 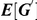 , 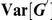	Observed 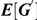 , 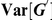
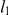 -vs- 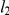	1.000, 2.000	1.018, 2.050	1.000, 0.0124	1.020, 0.0115
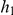 -vs- 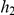	1.000, 2.000	1.015, 2.077	1.000, 0.0124	1.014, 0.0117

Theoretical and observed means and variances of 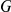 and 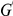 for the null comparisons in the yeast data set.

#### Between replicate comparisons of 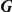 and 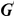 in the presence of a QTL

**Figure 3:**
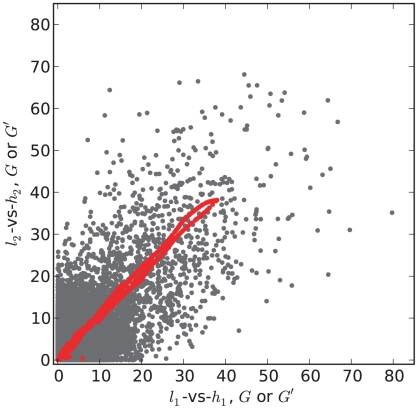
Comparison of 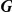 and 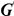 between technical replicates. The correspondence of raw 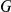 (black) and smoothed 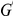 values (red) for different sequencing runs of the same low-vs-high bulks from the yeast data set.

In addition to tests of the null model, the design of the yeast experiment facilitates a between replicate comparison of 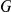 and 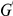 in the presence of QTLs. There are four possible low-vs-high comparisons; here we focus on two of those, 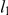-vs-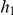 and 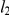-vs-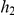. Figure 3 illustrates the relationships for 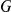 and 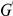 at each SNP for 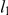-vs-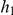 and 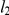-vs-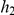. The between replicate correlation for 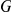 is 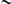
0.677, while that between 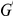 is 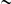
0.996. This illustrates the ability of the smoothing kernel to act as a low-pass filter on the 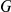 -statistic, filtering out the high-frequency noise associated with variation in read counts, while preserving the underlying signal of QTLs and increasing the repeatability of the analysis.

Using the false discovery rate approach outline above, we estimated cutoff values for 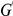 using a FDR of 0.01 based on both our theoretical results (equations 8 and 12) and the corresponding non-parametric estimators. For the parametric estimate we used the following parameter values: 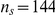, 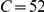, 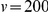. The estimated 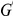 cutoff values are as follows: 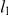-vs-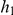 : 2.59 [parametric], 3.51 [non-parameteric]; 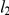-vs-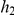 : 2.58 [parametric], 3.91 [non-parametric].

**Figure 4:**
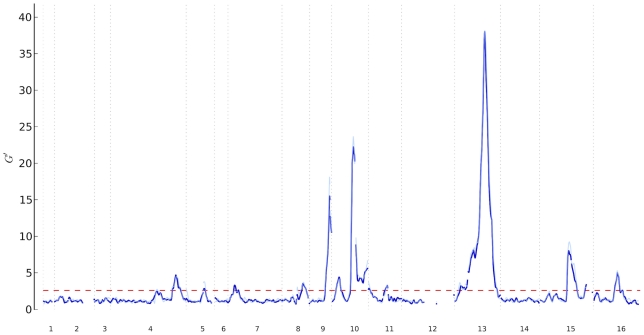
Yeast QTL Peaks. Chromosomal distributions of 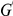 for the 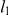-vs-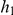 (dark blue) and 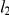-vs-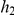 (light blue) data sets. The dashed red line indicates the estimated 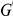 threshold corresponding to a FDR of 0.01. Regions above the red line are QTL regions; the highest point in each QTL region was called as the QTL peak.

Using the theoretical 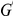 cutoff of 2.59 we find 7,845 SNPs have significant 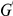 values for the 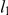-vs-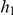 comparison, and 8,011 significant SNPs for the 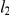-vs-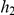 comparison, representing approximately 17% of the polymorphic sites. Nearly 38% of the significant sites are on chromosome XIII which appears to have multiple overlapping peaks leading to elevated 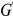 values across much of the chromosome. The number of significant sites shared between the replicates is 7,330. We identified 12 significant regions (QTLs) in the two replicates (Figure 4). The QTLs are nearly identical between the replicates except for a marginal QTL on chromosome 7, where one of the replicates is significant but the other is just short of significance. To assess the variability in QTL location we compared the distance between peaks (using the single largest peak in cases of multiple peaks per chromosome). The mean and median absolute distances between nine comparable QTL peaks from the two comparisons are 5.08 Kb and 4.97 Kb respectively. The root mean square deviation (RMSD) between comparable QTL peaks is 6.7 Kb. Using the RMSD as a measure of spread and applying the 3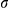 rule of thumb, a conservative confidence interval for QTL peak is 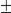
20 Kb (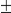
7.4 cM) around the observed peak. The size of this confidence interval is a function of read depth and SNP density, and *is a measure of variability in peak estimation due to sequencing only*. This confidence interval doesn't include variation that would arise from the bulking of segregants.

As will be described elsewhere, candidate genes corresponding to several of the major peaks in this analysis have been functionally validated to affect yeast colony morphology (J. Granek and P. Magwene, unpublished data).

## Discussion

The use of a test based on the 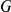 -statistic provides a straightforward framework for analyzing BSA-sequencing data. The 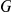 -statistic has several advantages over the use of allele frequency differences as the basis for QTL estimation (e.g. [11]). For example, as shown in the supporting information (Text S2), 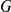 is expected to decrease much more rapidly around the causal site than bias in allele frequencies, implying narrower intervals of support around QTLs. Also in contrast to statistics based on the divergence of allele frequencies, 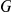 takes into account the strength of evidence related to sample size. This feature of the 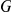 -statistic can also potentially complicate analyses, as variance in read depth contributes to variance in 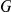 over relatively small spatial scales. However, as we show above, weighted averaging of 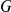 effectively smooths out ‘high frequency’ noise associated with sequencing variation.

### Bulk Size and Sequencing Considerations

Our simulations suggest that for the experimental design considered here using bulk sizes as large as 15–20% of the phenotyped segregant population increases power to detect causal QTLs despite the fact that this means relatively smaller allele frequency differences between bulks. This is due to tradeoffs between bulk-size, selection intensity, and the variance of allele frequencies under the hierarchical sampling. Consider, for example, a single locus with alleles 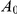 and 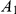, where the effect of 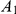 is additive and the two homozygotes differ by 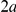 units on average. Assuming no segregation distortion, and an 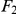 population generated from inbred lines, the change in the allele frequency of 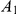 in the high bulk after truncation selection is approximately 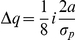 [30], [31] where 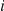 is the intensity of selection, and 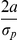 is the ‘standardized effect of the locus’ (these quantities can be related to the selection coefficient, 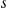, by 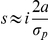). Given truncation selection on a normal distribution, the intensity of selection is given by 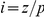 where 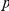 is the proportion of selected individuals and 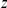 is the probability density function at the truncation point [31]. Since the intensity of selection increases at a rate much less than 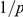 (e.g. see [31], Fig. 11.3), an 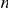-fold decrease in 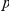 results in a much less than 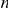-fold change in the intensity of selection. For example, let 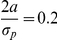 and consider truncation on the upper 20%, 10%, and 1%, of the phenotypic distribution. The increase in the frequency of 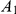 in the high bulk given these truncation points is approximately 3.5%, 4.4%, and 6.7% respectively (translating to allele frequency differences of 7%, 8.8%, and 13.4% in the two-bulk case). On the other hand, the variance of the realized frequencies of the alleles in each bulk is inversely proportional to bulk size (
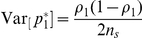
). Thus, a twenty-fold decrease in bulk size translates to less than a two-fold increase in allele frequency divergence, but a twenty-fold increase in the variance of allele frequencies. As long as average coverage, 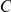, is moderate to large, the benefit of increasing 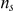 offsets the relatively smaller penalty resulting from a decrease in selection intensity. However, there is little benefit to increasing sequencing coverage beyond the size of the bulks.

Sequencing can introduce complications such as biases toward particular nucleotide calls; however in general this should effect both segregant bulks in the same direction. Due to the averaging affect of 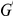, unless such biased sites are common over very large map distances they are unlikely to have substantial affects on results derived under our proposed framework. Similarly, a low percentage of mismapped reads or miscalled SNP calling are unlikely to be problematic for our framework, again because of the averaging affect of 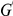. However caution should be exercised in genomic regions that are particularly problematic in this regard, such as repeat rich regions.

### Other Experimental Designs

In this paper we have focused on QTL mapping with an F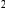 experimental design, but clearly our framework can be extended to other designs. Common alternatives include mapping populations produced by imposing one or more generations of inbreeding on an F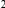, such as Recombinant Inbred Lines (RILs). The increased homozygosity of such populations should also be taken into consideration, as it increases the expected change in allele frequency due to selection but it also decreases the number of independent chromosomes that are sampled for a given number of selected individuals. Chromosomes in such RILs experience as much as twice the number of crossovers as do F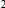 populations so the physical size of the smoothing window 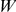 should be reduced to take this reduced linkage disequilibrium into account. Even greater reductions of linkage disequilibrium can be accomplished by an alternative design that imposes additional generations of random mating, rather than inbreeding, on an F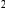, resulting in more precise localization of QTLs. Additional generations of outcrossing (beyond the F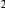) will likely magnify deviations of the null allele frequency from 0.5 owing to segregation distortion and/or inadvertent selection. This can be accommodated by application of formulas in Text S1 with 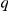 estimated from all sites within a genomic window.

Other experimental designs, such as backcrosses, will not have allele frequencies of 0.5. For these situations the null expected distributions of 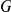 and 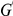 can be approximated using the equations presented in Text S1, although in this case it will be necessary to know the parental origin of the SNP alleles. Similarly, since 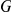 can be generalized to an arbitrary number of classes [12], one-tailed scenarios (e.g. [9]) involving comparison to either a theoeretical population or a random sampling of segregants can be addressed in this framework.

## Methods

### Sequencing of Yeast Bulks

To create the bulked DNA pools each segregant was grown overnight in liquid medium to saturation (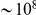 cells/ml) and equal volumes of each culture were mixed to form cell bulks. Genomic DNA was isolated from the cell bulks and single Illumina DNA sequencing libraries were prepared from each bulk, using standard protocols as described in [28]. Each bulk DNA pool was sequenced twice using 50 bp reads on an Illumina GAII sequencing instrument. Approximately 15 M reads were generated in each sequencing run. Reads were aligned to the yeast reference genome (obtained from the Saccharomyces Genome Database, January 2010) using the program BWA [32] and polymorphic sites were called using SAMtools [33]. For each sequencing run, SAMtools was used to create a pileup file giving the alleles at each polymorphic site, from which allele counts were derived using scripts written in Python.

## Supporting Information

Figure S1
**Simulations results for the null distribution of** 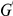 **based on 10,000 simulations with (** 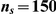 **,** 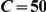 **,** 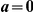 **).** The gray histogram represents the observed distribution of 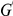, corresponding to Figure 1b. The dashed lines represent log-normal distributions estimated from theoretical expectation (red line) or via the non-parametric approach described in the text (black line). Both the parametric and non-parametric approaches provide good control of type I error (right tail of the distribution).(PDF)

Text S1 **Generalization of theoretical results to include segregation distortion.**
(PDF)

Text S2
**Miscellaneous information.** This file includes information on: 1) estimation of the parameters of a log-normal distribution from the expected mean and variance of a variable of interest; 2) the expected shape of the 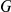 around at a QTL; and 3) A summary table of expected and observed means and variances of 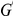 based on simulations of the null hypothesis (no QTL).(PDF)
