# Supplementary figures and images for: The Statistics of Bulk Segregant Analysis Using Next Generation Sequencing
Source: PLoS Comput Biol. 2011 Nov 3;7(11):e1002255. doi: 10.1371/journal.pcbi.1002255 (PMC3207950; doi:10.1371/journal.pcbi.1002255)

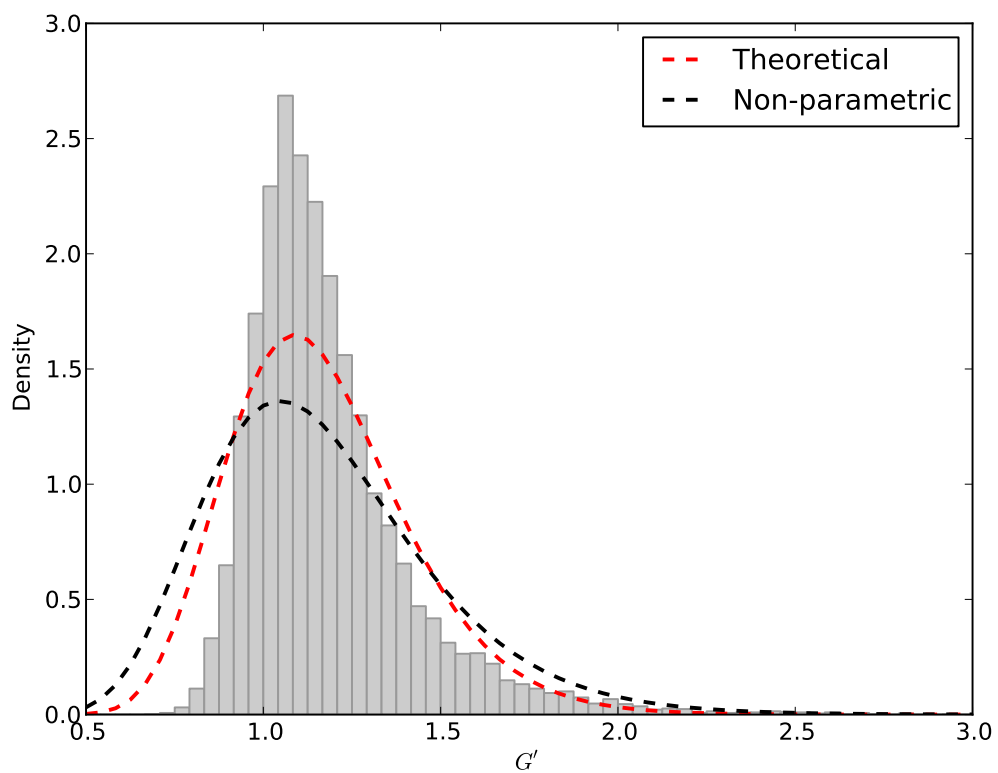

Supplement: Figure S1 — Simulations results for the null distribution of based on 10,000 simulations with ( , , ). The gray histogram represents the observed distribution of , corresponding to Figure 1b. The dashed lines represent log-normal distributions estimated from theoretical expectation (red line) or via the non-parametric approach described in the text (black line). Both the parametric and non-parametric approaches provide good control of type I error (right tail of the distribution). (PDF) [file pcbi.1002255.s001.pdf]
