# Supplementary material for: The Statistics of Bulk Segregant Analysis Using Next Generation Sequencing
Source: PLoS Comput Biol. 2011 Nov 3;7(11):e1002255. doi: 10.1371/journal.pcbi.1002255 (PMC3207950; doi:10.1371/journal.pcbi.1002255)
Supplement: Text S1 — Generalization of theoretical results to include segregation distortion. (PDF) [file pcbi.1002255.s002.pdf]

Consider the experimental design with an F2 population consisting of N individuals, each of which is measured for phenotype. A set of $n_S$ diploid individuals from the low end of the distribution and a set of $n_S$ individuals from the high end are collected. DNA is combined in equal amounts from individuals within each bulk, followed by sequencing at average coverage C per SNP. For each SNP, the data is four allele counts that can be summarized in a 2 x 2 table (Table 1 of main paper). The observed allele frequency in the Low bulk is $p_1 = n_3 / (n_1 + n_3)$. The observed allele frequency in the High bulk is $p_2 = n_4 / (n_2 + n_4)$. With this notation, the G-statistic for a snp is:

$$G = 2\sum_{i=1}^{4} n_i Ln[\frac{n_i}{n_i^*}] \tag{1}$$

with $n_i^*$ as the 'expected value' for count i. The null hypothesis is that there is no QTL close to our SNP. This implies the standard "expected counts" for a 2 x 2 contingency table, e.g. $n_1^* = (n_1 + n_2)(n_1 + n_3)/(n_1+n_2+n_3+n_4)$.

For the analytical calculations below, we approximate G with a second order polynomial expansion of equation (1) around the expected values for $n_1$, $n_2$, $n_3$, and $n_4$. Assuming equal (average) sequencing coverage of each bulk and the null hypothesis is correct, $E[n_1] = E[n_2] = q\,C$ and $E[n_3] = E[n_4] = (1-q)\,C$. The second order Taylor expansion is:

$$G \approx \frac{[(1-q)(n_2 - n_1) + q(n_3 - n_4)]^2}{2Cq(1-q)} \tag{2a}$$

If we further assume no segregation distortion (q=0.5), then $E[n_1] = E[n_2] = E[n_3] = E[n_4] = C/2$ and

$$G \approx \frac{[n_1 - n_3 - n_2 + n_4]^2}{2C} \tag{2b}$$

In contrast to the usual G-test (SOKAL and ROHLF 2000, ch 17), we do not expect that G will generally follow a chi-square distribution (1 d.f.) in the present situation. The counts in table 1 are determined by two distinct rounds of sampling. The first sample is the 2 $n_S$ chromosome sets that constitute each bulk (assuming diploid inheritance). Second, there is random variation in the number of reads per allele within each bulk due to the stochastic nature of next-generation sequencing. The two rounds of sampling inflate the mean and variance of G relative the chi-square with 1 d.f. even when the null hypothesis is true (there is no QTL). Given the magnitude of the QTL effect, the size of the F2 population (N), and the number selected from each tail ($n_S$), there is an expected frequency for the low allele ($A_0$) in each bulk (denoted $\theta_L$ and $\theta_H$, respectively). If there is no segregation distortion and $A_1$ increases phenotype then we expect $\theta_H < 0.5 < \theta_L$. If the QTL contributes additively to other sources of variation in phenotype, then $\theta_L$ and $\theta_H$ are simple functions of allelic effect and $n_S/N$ (see KIMURA and CROW 1978).

The actual frequency of $A_0$ in each bulk ($q_L^*$ and $q_H^*$) differ from $\theta_L$ and $\theta_H$ due to sampling (each bulk contains 2 $n_S$ alleles). We assume that this sampling is binomial. The actual estimated frequencies ($q_L$ and $q_H$) differ from $q_L^*$ and $q_H^*$ because of random variation in read

number. We assume that the read numbers for each bin of Table 1 are conditionally Poisson distributed:

$E[n_1 \ |q_L^*] = Var[n_1 \ |q_L^*] = Cq_L^*$ $\qquad$ $E[n_2 \ |q_H^*] = Var[n_2 \ |q_H^*] = Cq_H^*$

$E[n_3 \ |q_L^*] = Var[n_3 \ |q_L^*] = C(1-q_L^*)$ $\qquad$ $E[n_4 \ |q_H^*] = Var[n_4 \ |q_H^*] = C(1-q_H^*)$

and

$$E[q_L^*] = \theta_L, \qquad Var[q_L^*] = \frac{\theta_L(1-\theta_L)}{2n_S}$$

$$E[q_H^*] = \theta_H, \qquad Var[q_H^*] = \frac{\theta_H(1-\theta_H)}{2n_S}$$

Under the null hypothesis: $\theta_L$ and $\theta_H$ = q. To calculate the expected value for G, we need the expected values of counts:

$$E[n_1] = E[n_2] = Cq, \quad E[n_3] = E[n_4] = C(1-q)$$

and the expected values for squares and cross products:

$$E[n_1^2] = E[Cq_L^* + (Cq_L^*)^2] = Cq + C^2(q^2 + q(1-q)/(2n_S)) = E[n_2^2]$$

$$E[n_3^2] = E[C(1-q_L^*) + (C(1-q_L^*))^2] = C(1-q) + C^2((1-q)^2 + q(1-q)/(2n_S)) = E[n_4^2]$$

$$E[n_1 n_2] = (Cq)^2$$

$$E[n_1 n_3] = E[Cq_L^* C(1-q_L^*)] = C^2(q - q^2 - q(1-q)/(2n_S))$$

$$E[n_1 n_4] = C^2 q(1-q)$$

$$E[n_2 n_3] = C^2 q(1-q)$$

$$E[n_2 n_4] = E[Cq_H^* C(1-q_H^*)] = C^2(q - q^2 - q(1-q)/(2n_S))$$

$$E[n_3 n_4] = (C(1-q))^2$$

Substituting into equation (2a) and then simplifying, we find that

$$\text{E[G]} \approx 1 + \frac{C}{2n_S} \qquad (3)$$

The expectation does not depend on q.

To calculate the variance, we need 3rd and 4th order moments of the counts. Symmetry implies that we can use the moments involving $n_1$ and $n_3$ to deduce moments involving $n_2$ and $n_4$. After substantial algebra, we find that:

$$Var[G] \approx \frac{4n_S(n_S + C)(1-2q)^2 + C^2(1 + q(1-q)(8n_S - 6))}{16 n_S{}^3 q(1-q)} +$$

$$\frac{1-3q(1-q)}{Cq(1-q)} + \frac{C(1+4q(1-q)(n_S-1))}{2n_S{}^2 q(1-q)} + \frac{3 + q(1-q)(4n_S - 10)}{2n_S \ q(1-q)} \qquad (4)$$

which can be written as

$$\frac{8n_S^{\,3}(1-3q(1-q))+4Cn_S^{\,2}(7+8(n_S-3)q(1-q))+4C^2n_S\ (3+4(2n_S-3)q(1-q))+C^3(1+(8n_S-6)q(1-q))}{16Cn_S^{\,3}q(1-q)}$$

With no segregation distortion (q = 0.5), the equation for the variance simplifies:

$$Var[G]\approx 2+\frac{1}{2C}+\frac{1+2C}{n_S}+\frac{C^2(4n_S-1)}{8n_S^{\,3}} \tag{5}$$

These equations predict convergence on chi-square-1 when $n_S >> C >> 1$. Then E[G] → 1 and Var[G] → 2.

An alternative and perhaps more intuitive way to obtain $Var[G]$ is to note that variance in G has two sources. There is sampling of the individuals in each bulk and read number variation. Using the standard rule for calculating the variance of a conditional random variable:

$$Var[G]=E[Var[G\ |q_L^{\,*},q_H^{\,*}]]+Var[E[G\ |q_L^{\,*},q_H^{\,*}]] \tag{6}$$

$E[G\ |q_L^{\,*},q_H^{\,*}]$ is obtained by the steps used above to derive E[G] except without averaging over the distributions of realized allele frequencies within bulks ($q_L$* and $q_H$*). The relevant expression is:

$$E[G\ |q_L^{\,*},q_H^{\,*}]\approx\frac{2q^2+C(q_L^{\,*}-q_H^{\,*})^2+(1-2q)(q_L^{\,*}+q_H^{\,*})}{2q(1-q)} \tag{7}$$

This reduces to $E[G\ |q_L^{\,*},q_H^{\,*}]\approx 1+2C(q_L^{\,*}-q_H^{\,*})^2$ if q = 0.5. The variance (across replicated experiments) for this average is

$$Var[E[G\ |q_L^{\,*},q_H^{\,*}]]=\frac{Var[C(q_L^{\,*}-q_H^{\,*})^2+(1-2q)(q_L^{\,*}+q_H^{\,*})]}{4q^2(1-q)^2} \tag{8}$$

Binomial sampling of chromosomes to obtain bulks implies:

$$Var[E[G\ |q_L^{\,*},q_H^{\,*}]]=\frac{4n_S(n_S+C)(1-2q)^2+C^2(1+q(1-q)(8n_S-6))}{16n_S^{\,3}q(1-q)} \tag{9}$$

This reduces to $\frac{C^2(4n_S-1)}{8n_S^{\,3}}$ if q = 0.5. This is the first term of equation (4) above. The other three terms are $E[Var[G\ |q_L^{\,*},q_H^{\,*}]]$.

The conditional partitioning is useful mainly to calculate the covariance of G at distinct sites. Let $Cov[G_A,G_B]$ denote the covariance of G values for at two linked sites A and B, which should be positive for closely linked SNPs. Without recombination, neighboring SNPs will share the same values for $q_L$* and $q_H$*. To consider recombination, let $x_1$, $x_2$, $x_3$, and $x_4$ denote the haplotype frequencies of AB, Ab, aB, and ab for two linked snps in the low bulk. $y_1$, $y_2$, $y_3$, and $y_4$ are the corresponding haplotype frequencies in the high bulk. The covariance of G values

from snps A and B can be usefully partitioned by conditioning on observed haplotype frequencies:

$$Cov[G_A, G_B] = E[Cov[G_A, G_B | x, y]] + Cov[E[G_A | x, y], E[G_B | x, y]] \quad (10)$$

This partitioning is useful because $Cov[G_A, G_B | x, y]$ should be zero. As long as counts at sites A and B are determined by distinct (and independently sampled) sequence reads, then $Cov[G_A, G_B]$ depends on only the sampling of individuals into bulks. Assuming (importantly) that q is the same for both snps,

$$Cov[E[G_A | x, y], E[G_B | x, y]] = \quad (11)$$

$$Cov[\frac{C(x_1 + x_2 - y_1 - y_2)^2 + (1-2q)(x_1 + x_2 + y_1 + y_2)}{2q(1-q)}, \frac{C(x_1 + x_3 - y_1 - y_3)^2 + (1-2q)(x_1 + x_3 + y_1 + y_3)}{2q(1-q)}]$$

Under the null hypothesis, the moments for haplotype frequencies can be calculated from the multinomial distribution. The expected values depend on the nature of segregation distortion, but a sensible scheme is:

$E[x_1] = E[y_1] = q - r_{AB}/2$,

$E[x_2] = E[y_2] = r_{AB}/2$

$E[x_3] = E[y_3] = r_{AB}/2$

$E[x_4] = E[y_4] = 1 - q - r_{AB}/2$.

Noting that observed haplotype frequencies in the low bulk are independent of the frequencies in the high bulk, and using the moments for x and y up to fourth order, and performing a substantial algebraic simplification, we find that

$$Cov[(x_1 + x_2 - y_1 - y_2)^2, (x_1 + x_3 - y_1 - y_3)^2] = \frac{(2q(1-q) - r_{AB})(1 + (8n_S - 6)q(1-q) + (4n_S - 1)r_{AB})}{8n_S^{\ 3}}$$

$$Cov[(x_1 + x_2 - y_1 - y_2)^2, (x_1 + x_3 - y_1 - y_3)] = \frac{(1-2q)(2q(1-q) - r_{AB})}{4n_S^{\ 2}}$$

$$Cov[(x_1 + x_2 - y_1 - y_2)\ , (x_1 + x_3 - y_1 - y_3)^2] = \frac{(1-2q)(2q(1-q) - r_{AB})}{4n_S^{\ 2}}$$

$$Cov[(x_1 + x_2 + y_1 + y_2)\ , (x_1 + x_3 + y_1 + y_3)] = \frac{4q(1-q) - r_{AB}(2 - 4q + r_{AB})}{4n_S}$$

Substituting these into eq (11), we find that

$$Cov[G_A, G_B] = \quad Var[E[G | q_L^{\ *}, q_H^{\ *}]](\frac{2q(1-q) - r_{AB}}{2q(1-q)}) +$$

$$r_{AB}(\frac{2n_S^{\ 2}(1-2q)^2(4q - r_{AB}) + C^2(4n_S - 1)(2q(1-q) - r_{AB})}{32q^2(1-q)^2 n_S^{\ 3}}) \quad (12)$$

This reduces to

$$Cov[G_A, G_B] = \frac{C^2(4n_S - 1)}{8{n_S}^3}(1 - 2r_{AB})^2 \quad (13)$$

if q = 0.5.

**Calculation of G'—**Averaging G values across neighboring SNPs is sensible because the real signal—divergence in allele frequency between bulks—is conserved between closely linked sites but random noise due to variable sequencing read coverage is not. We suggest a weighted average test statistic for each SNP:

$$G' = \sum_{j\ in\ W} k_j G_j \quad (14)$$

with

$$k_j = (1 - {D_j}^3)^3 / S_W \quad (15)$$

where $D_j$ is standardized to have value 0 at the focal position and value 1 at the edge of the window. $S_w$ is the sum of $(1 - {D_j}^3)^3$ for all snps in W.

For all derivations below, we assume q = 0.5. The null expectation of G' is given by equation (3). The variance of G' depends on the variance of individual G values (equation 5) and the covariance between SNPs (equation 13) within a window:

$$Var[G'] = (2 + \frac{1}{2C} + \frac{1 + 2C}{n_S} + \frac{C^2(4n_S - 1)}{8{n_S}^3})(\sum_{j\ in\ W} {k_j}^2) + \sum_{j\ in\ W} \sum_{i \neq j} \frac{C^2(4n_S - 1)}{8{n_S}^3}(1 - 2r_{ij})^2 k_i k_j \quad (16)$$

where i indexes all SNPs other than j contained within the window.

Cited in Supplementals:

KIMURA, M., and J. F. CROW, 1978 Effect of overall phenotypic selection on genetic change at individual loci. Proc. Natl. Acad. Sci. USA **75:** 6168-6171.

SOKAL, R. R., and F. J. ROHLF, 2000 *Biometry*. Freeman and company, New York.
