# Supplementary material for: The Statistics of Bulk Segregant Analysis Using Next Generation Sequencing
Source: PLoS Comput Biol. 2011 Nov 3;7(11):e1002255. doi: 10.1371/journal.pcbi.1002255 (PMC3207950; doi:10.1371/journal.pcbi.1002255)
Supplement: Text S2 — Miscellaneous information. This file includes information on: 1) estimation of the parameters of a log-normal distribution from the expected mean and variance of a variable of interest; 2) the expected shape of the around at a QTL; and 3) A summary table of expected and observed means and variances of based on simulations of the null hypothesis (no QTL). (PDF) [file pcbi.1002255.s003.pdf]

Supporting Information:
The Statistics of Bulk Segregant Analysis Using Next Generation Sequencing

Paul M. Magwene, John H. Willis, John K. Kelly

Derivation of Log-Normal Distribution from $E[G']$ and Var$[G']$

If G' is log-normally distributed, then:

$$\begin{aligned} E[G'] &= e^{\mu+\frac{1}{2}\sigma^2} \\ \mathrm{Var}[G'] &= (e^{\sigma^2}-1)e^{2\mu+\sigma^2} \end{aligned}$$

Given $E[G']$ and Var$[G']$ (see text), we can estimate the parameters of the log-normal distribution as:

$$\begin{aligned} \mu &= \ln(E[G']) - \frac{1}{2}\left(1+\frac{\mathrm{Var}[G']}{E[G']^2}\right) \\ \sigma^2 &= \ln\left(1+\frac{\mathrm{Var}[G']}{E[G']^2}\right) \end{aligned}$$

Expected shape of G around a causal SNP

Assume the parental genotypes at two loci are AB and ab, and that there is a single causal allele, A, at the A locus. If p_A is the frequency of allele A in a bulk of F_2 segregants, the expected allele frequency of B is $p_B = p_A(1-2r_{AB}) + r_{AB}$, where r_{AB} is the recombination rate between A and B. The frequency of allele b is $p_b = 1 - p_B$. Ignoring sampling variation, and assuming the expected frequency of each allele is 0.5, G is proportional to:

$$p_{B,l}\ln p_{B,l} + p_{b,l}\ln p_{b,l} + p_{B,h}\ln p_{B,h} + p_{b,h} + \ln p_{b,h} + 2\ln 2$$

If A is fixed in a bulk, then $p_B = 1 - r_{AB}$ and $p_b = r_{AB}$. If we assume that the allele A is fixed in one bulk, and at the expected frequency in the other bulk than G should be proportional to:

$$r_{AB}\ln r_{AB} + (1-r_{AB})\ln(1-r_{AB}) + \ln 2$$

A scaled and normalized representation of allelic bias ($p_B - 0.5$) and the corresponding normalized G around a causal SNP are illustrated in Figure 1, with respect to map distance (after converting map distance to recombination rate using Haldane's formula).

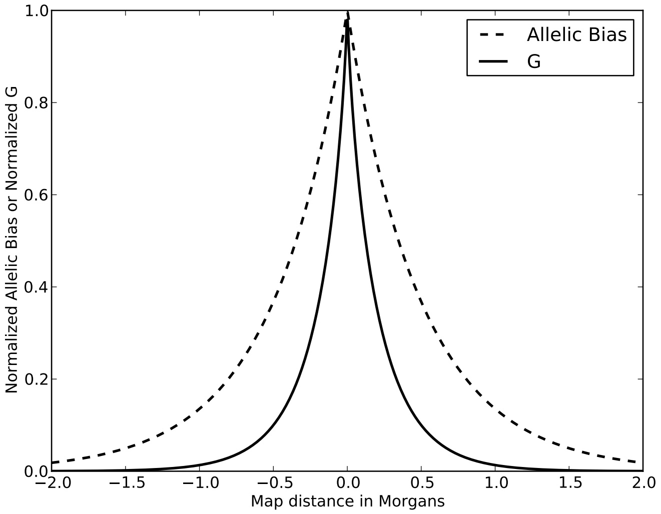

Figure 1: The allelic bias (dashed line) and G statistic around a causal SNP, not accounting for sampling variation.

One interesting feature to note is that the full-width at half maximum (FWHM) for G is significantly smaller than that of allelic bias, indicating that the signal for G falls of more rapidly around the causal SNP than the comparable signal for allelic bias. Assuming sufficient SNP density, this is a useful aspect of G because it implies relatively smaller support intervals.

Supplementary Tables

nS	C	Mean G' Observed	Predicted	Variance of G' Observed	Predicted
20	10	1.38	1.25	0.14	0.13
20	20	1.56	1.50	0.42	0.45
20	40	2.05	2.00	1.66	1.71
20	100	3.57	3.50	10.12	10.53
50	25	1.29	1.25	0.11	0.13
50	50	1.52	1.50	0.41	0.45
50	100	2.02	2.00	1.60	1.73
50	250	3.54	3.50	9.88	10.61
100	50	1.27	1.25	0.11	0.13
100	100	1.51	1.50	0.41	0.45
100	200	2.02	2.00	1.57	1.73
100	500	3.50	3.50	9.53	10.64
200	100	1.27	1.25	0.11	0.13
200	200	1.51	1.50	0.41	0.45
200	400	2.02	2.00	1.57	1.73
200	1000	3.51	3.50	9.73	10.65

Table 1: A summary of repeated simulations of the null hypothesis with 100,000 replicates per parameter set. For all simulations, the window size was 20 cM with 10 SNPs per cM. The mean coverage per bulk, C, was varied from half n_s to five times n_s for each value of n_s.
